# Characteristics of Older Veterans Enrolled in the Illinois Medical Cannabis Patient Program

**DOI:** 10.1093/geroni/igaa057.1546

**Published:** 2020-12-16

**Authors:** Julie Bobitt, Hyojung Kang

**Affiliations:** 1 University of Illinois at Urbana-Champaign, Champaign, Illinois, United States; 2 University of Illinois - Urbana/Champaign, Champaign, Illinois, United States

## Abstract

Veterans often struggle with disabling conditions such as chronic pain and post-traumatic stress disorder (PTSD) that tend to worsen as they age. Common treatments for these conditions include the use of opioids and benzodiazepines, yet these medications tend to have unwanted side effects and can even result in addiction. While cannabis use in the US has increased significantly over the past decade, research regarding the risks and benefits is mixed and a growing number of research studies have highlighted the benefits of taking cannabis for medical purposes. While previous studies have looked at cannabis use in older adults and in Veterans over 18, no research has looked at cannabis use specifically in older Veterans. We surveyed adults age 60 and over who were enrolled in the Illinois Medical Cannabis Patient Program as of September 2019. We collected demographics, reason for enrolling in the medical cannabis program, history of cannabis, opioids, and/or benzodiazepines use, and health outcomes. Of 3,768 responses, 593 were Veterans. Older Veterans in our study were predominately male (92.1%), reported using cannabis primarily for pain (80.1%), PTSD or emotional health problems (50.1%) (i.e. anxiety and depression), and reported that cannabis use has positively impacted their quality of life (89.4%), health outcomes (81.9%), pain (86%), and sleep quality (77.1%). Understanding why older Veterans use cannabis and the outcomes they experience from cannabis use can inform state and federal policy makers and enhance clinical care practices.

